# Clinical and functional outcome measures in LAMA2-related muscular dystrophy and SELENON-related myopathy; a 1.5-year natural history study

**DOI:** 10.1177/22143602261465467

**Published:** 2026-08-01

**Authors:** Elisabeth C. M. de Laat, Jan T. Groothuis, Karlijn Bouman, Saskia L. S. Houwen – van Opstal, Corrie E. Erasmus, Nicol C. Voermans

**Affiliations:** 1 Department of Neurology, Radboudumc Research Institute for Medical Innovation, Donders Institute for Brain, Cognition and Behaviour, 6034 Radboud University Medical Center, Nijmegen, The Netherlands; 2 Department of Rehabilitation, Donders Institute for Brain, Cognition and Behaviour, 6034 Radboud University Medical Center, Nijmegen, The Netherlands; 3 Department of Paediatric Neurology, Donders Institute for Brain, Cognition and Behaviour, Amalia Children’s Hospital, 6034 Radboud University Medical Center, Nijmegen, The Netherlands; 4 Department of Rehabilitation, Donders Institute for Brain, Cognition and Behaviour, Amalia Children’s Hospital, 6034 Radboud University Medical Center, Nijmegen, The Netherlands

**Keywords:** LAMA2-related muscular dystrophy, SELENON(SEPN1)-related myopathy, outcome measures, natural history, trial readiness

## Abstract

**Background:**

LAMA2-related muscular dystrophy (LAMA2-MD) and SELENON-related myopathy (SELENON-RM) are rare congenital muscle diseases characterized by slowly progressive proximal muscle weakness, spinal rigidity and respiratory insufficiency. The LAST STRONG study is a natural history study to identify suitable outcome measures and reach trial readiness for LAMA2-MD and SELENON-RM.

**Methods:**

Patients had four visits over 1.5-years. Assessments included neurological examination, hand-held dynamometry (HHD), functional assessments (Motor Function Measurements(MFM)-20/32, graded and timed function tests), accelerometry, and questionnaires on quality of life, activities and participation, pain, and fatigue.

**Results:**

A total of 27 LAMA2-MD (21 years, range 3-50; 9 males) and 11 SELENON-RM (20 years, range 3-42; 8 males) patients were included. In LAMA2-MD, mean HHD scores for neck extensors, biceps brachii, quadriceps, foot plantar flexors and handgrip strength increased (all p < 0.02). In SELENON-RM, biceps brachii and foot plantar flexors increased (p < 0.01). In LAMA2-MD, functional assessments did not change significantly. In SELENON-RM, MFM-20/32 total score and domain 1 decreased (p < 0.05), 6-Minute Walk Test (6MWT) decreased (p < 0.01) and 10-Meter Walk Test (10MWT) increased (p < 0.01). Accelerometry showed a significant change in moderate activity in LAMA2-MD (p <0.01), no changes were observed in SELENON-RM.

**Discussion:**

Over 1.5 years, LAMA2-MD remained mostly stable, while SELENON-RM showed minimal functional decline on select outcome measures. Both cohorts showed HHD increases, likely reflecting age-related development. Most measures did not capture disease progression, however MFM-20/32, 6MWT and 10MWT showed small significant changes in SELENON-RM, suggesting potential trial endpoints.

## Introduction

Laminin-α2-related muscular dystrophy (LAMA2-MD) and selenoprotein N-related congenital myopathy (SELENON-RM) are rare congenital muscle diseases with similar clinical features and an estimated prevalence of 8.3 and 0.5 per 1,000,000 individuals world-wide, respectively.^1,2^ LAMA2-MD is characterized by a clinical disease spectrum ranging from a severe early-onset congenital muscular dystrophy type 1A to a milder, later-onset limb-girdle-type muscular dystrophy.^2,3^ Key clinical features of both LAMA2-MD and SELENON-RM are delayed motor development, slowly progressive axial and proximal muscle weakness, early-onset spinal rigidity, scoliosis, and respiratory insufficiency.^2^ Additionally, LAMA2-MD patients may suffer from epileptic seizures and demonstrate abnormal (increased) T2 signal on magnetic resonance imaging (MRI) related to the water content of the myelin in the brain.^4^ Both LAMA2-MD and SELENON-RM are diagnosed by recessive pathogenetic loss-of-function variants in the *LAMA2* or *SELENON* gene. Variants in these genes have distinct cellular consequences. *LAMA2* variants cause laminin-α2 deficiency, which disrupts laminin-211 function at the muscle basement membrane compromising membrane instability. This results in impaired fiber adhesion and regeneration, increased cell loss, and progressive muscle weakness with fibrosis and fatty replacement.^5^ Variants in the *SELENON* gene lead to increased oxidative stress, disrupted calcium homeostasis, impairing excitation-contraction coupling and muscle fiber maintenance. This results in reduced muscle endurance, limited regenerative capacity, and progressive weakness predominantly affecting axial and respiratory muscles.^6^ Currently, no curative treatments exist, although preclinical studies are ongoing.^7–11^

LAMA2-MD and SELENON-RM are both characterized by significant variability in symptoms and clinical disease course. While previous research, including retrospective studies, has contributed to understanding LAMA2-MD and SELENON-RM, detailed prospective natural history data including rate of progression remain unclear.^12–14^

Identifying sensitive and relevant clinical and functional outcome measures is essential for assessing disease progression, improving patient care and reaching trial readiness. The LAST STRONG (LAMA2-MD and SELENON-RM To Study Trial Readiness, Outcome measures and Natural history) study is a 1.5-year longitudinal natural history study investigating multiple outcome measures in LAMA2-MD and SELENON-RM.^15,16^ The present manuscript focuses exclusively on clinical and functional outcome measures obtained during this 1.5-year follow-up, representing a subset of the overall dataset. Findings from the cardiac, respiratory and bone quality assessments have been reported separately.^17–19^ Baseline findings of the LAST STRONG have been previously published in two cross-sectional analyses of the same cohort, indicated that the motor function measurement (MFM)-20/32 is both suitable and feasible for evaluating disease severity in both LAMA2-MD and SELENON-RM across the lifespan.^20,21^ The 6-minute walk test (6MWT) is considered suitable to measure disease severity in ambulant patients. Further, time to rise from floor is of interest as it shows high variability among patients, its often reported difficult to perform, and has been established as a meaningful outcome in Duchenne muscular dystrophy (DMD).^22^ To ensure a more definite selection of sensitive and relevant outcome measures, longitudinal data collection is needed.

Hereby, we present the 1.5-year follow-up of the clinical and functional outcome measures of the LAST STRONG study. The aim of this study is to assess the natural course of LAMA2-MD and SELENON-RM in a 1.5-year follow-up period, to identify relevant and sensitive clinical and functional outcome measures that can be used to measure progression of the disease and therefore, used as a basis for future clinical trials. Given that LAMA2-MD and SELENON-RM are both ultrarare and have similar clinical phenotypes, they are well suited to be assessed in a *basket natural history study*. This design enables a comparison of shared characteristics and distinct differences in disease progression and underlying biology. This can provide valuable insights into their similarities and differences.

## Methods

### Study design and population

Patients with LAMA2-MD and SELENON-RM were recruited non-selectively and consecutively between August 2020 and May 2022 as part of the LAST STRONG Study. An elaborate description of the protocol is available elsewhere.^15^ All patients and/or their legal representatives provided informed consent prior to their inclusion in the study, and procedures were performed in the accordance with the ethical standards in the declaration of Helsinki. Inclusion criteria were a genetic confirmation of LAMA2-MD or SELENON-RM by two recessive (likely) pathologic mutations in the *LAMA2* or *SELENON (SEPN1)* gene, or typical clinical and histological alterations combined with genetic confirmation in a first degree relative. Exclusion criteria were an insufficient proficiency in the Dutch language.

All patients were invited for four visits over a period of 1.5 years, with each visit occurring at six-months intervals. If patients did not wish or were not able to visit our hospital, they were offered to participate through home visits, during which the evaluator performed the assessments in person. The protocol included a standardized medical history examination, standard neurological examination, muscle strength tests, a variety of functional measurements, accelerometery, and questionnaires.

### Neurological examination

Each visit started with an interview (K.B.) on medical history, current health status and devices. Furthermore, patients underwent a standard neurological examination by one assessor (K.B.). Additionally, facial weakness, reflexes, muscle tone and dysmorphic features were assessed by two independent assessors (K.B. and C.E. or N.V). The passive range of motion of the elbow, wrist, hip, knee and ankle joints was assessed by goniometry (K.B.).^23^

### Muscle strength

Muscle strength was assessed using the Medical Research Council (MRC) grading scale of the following muscles: neck flexor, neck extensor, sternocleidomastoid, trapezius, deltoid, biceps brachii, triceps brachii, wrist extensor, wrist flexor, finger extensor, finger flexor, finger spreader, gluteus maximum, iliopsoas, quadriceps, hamstrings, foot dorsiflexor, foot plantarflexor, extensor hallucis longus and toe flexor muscles. Further, muscle strength was measured using handheld dynamometry (HHD) (Citec, CT3002) in muscles with a MRC scale of ≥ 4.^24,25^ MRC sum score was calculated by adding the scores for the deltoid, biceps brachii, wrist extensors, quadriceps and foot dorsiflexors on both the left and right sides.

### Functional measurements

Motor function was assessed using the MFM-20/32, a scale used in patients with neuromuscular disorders that consists of 20 or 32 items in three different dimensions (standing position and transfers, axial and proximal motor function, and distal motor function.^26,27^ Furthermore, graded and timed function tests (GTFTs) consisting of 30 seconds sit to stand test, climb 4 stairs, descend 4 stairs, rise from floor, timed up and go test (TUG), 10-meter walk test (10MWT) and 6MWT were performed.^28–30^

All functional measurements were adapted to the patient’s age and functional abilities. If patients were unable to perform (part of) the GTFTs during any follow-up visits, the corresponding data were recorded as missing, as no maximum value is defined for these outcome measures.

### Accelerometery

Daily activity in the home situation during 7 consecutive days was measured using an accelerometer (GENEActiv Original, Activinsights Ltd, 87.5 Hz sampling) on the nondominant wrist after each study visit. The gravity-subtracted sum of vector magnitudes (SVMgs) was used as the activity measure. All data were subsequently analysed using MATLAB (version R2022a, MathWorks). Daily activity was expressed as counts per day and the percentage of light, moderate, vigorous and sedentary activity corrected for age.^31,32^

### Questionnaires

Patients and/or their legal representative(s) completed age-adapted questionnaires. The selection of questionnaires has been described in the published study protocol, and consisted of the following^15^:

1. Pediatric quality of life inventory (PedsQL) (Generic Core Scale, Neuromuscular Module (NMM) and Multidimensional Fatigue Scale (MFS)) were used to assess quality of life across multiple domains (patients between 2 and 17 years old). A higher score indicates a better Health-Related Quality of Life (HRQOL).^33–35^
2. Research and development-36 (RAND-36) was used to assess quality of life with 36 items across eight dimensions (age ≥ 18 years).^36^ A higher score indicates a higher quality of life.
3. Individualized neuromuscular quality of life (INQoL) was used to measure the impact of symptoms on quality of life specifically for individuals with neuromuscular disorders (age ≥ 18 years).^37^ A higher subscale score indicates a greater impact of the disease on the patient’s symptoms or life domains.
4. McGill pain questionnaire was used to assess location, level and characteristics of experienced pain (age ≥ 12 years).^38^ Where the higher the pain score, the greater the pain.
5. Wong-Baker faces pain rating scale is a visual analogue scale, consisting of a series of faces ranging from smiling to crying, used to assess and communicate pain intensity (age ≥ 2 years).^39^ A higher score indicates more pain.
6. Checklist individual strength (CIS) was used to assess fatigue using the following four subscales: subjective tiredness, concentration, motivation and, physical activation (age ≥ 18 years).^40,41^ A score of > 27 indicates an abnormal fatigue.
7. Activity limitations questionnaire (ACTIVLIM) was used to assess the ability to perform 22 activities of daily life on a three-point scale, from impossible to easy (age ≥ 6 years).^42,43^
8. Impact on participation and autonomy (IPA) was used to evaluate the participation and autonomy in daily life (age ≥ 18 years).^44^ Higher scores indicate more barriers to participation and autonomy or greater problem experience.
9. Egen Klassification version 2 (EK2) was used to assess the functional ability of activity in daily life among non-ambulatory Duchenne muscular dystrophy patients (age ≥ 12 years and sufficient understanding of the English language).^45^

### Statistical analysis

Statistical analyses were performed with R version 4.1.3 (R Core Team, 2022). Normality was tested, and data was checked for homoscedasticity. Continuous variables were expressed as median (interquartile range [IQR]), unless otherwise stated. Categorical data were given as a percentage. Simple descriptive statistics were performed.

Linear mixed models were used to analyze changes in disease progression over time. If patients only had one single visit with a score for a specific outcome measure, they were excluded from the data analysis of that specific outcome, as longitudinal data was required for analysis. The outcome measure (e.g. MFM total score or domain scores) was specified as the dependent variable, with visit (time) as the fixed-effect predictor. The level of statistical significance was set at *p*≤ 0.05.

### Standard protocol approvals, registrations, and patient consensus

The protocol has been approved by the medical ethical reviewing committee of Region Arnhem-Nijmegen (NL-number NL64269.091.17, dossier number 2017-3911; 8 July 2020) and was registered at clinicaltrials.gov (NCT4478981). From all patients, or in case of children their parents or legal guardian, informed consent was obtained for participating in our study. A consent-to-disclose form was signed by any identifiable person.

### Data availability

The data that support the findings of this study are available from the corresponding author on reasonable request.

## Results

### Patients’ characteristics

Twenty-seven LAMA2-MD patients (9 males, 18 females) with a median age of 21 (range 3 - 50) years were included. Eight patients were ambulant at the time of inclusion. Four patients did not have complete follow-up due to cardiorespiratory arrest (two hospital visits), burden of participation (one hospital visit, two home visits), personal circumstances (three home visits) and, late inclusion (one hospital visit). Seven patients had a feeding tube at baseline, this increased to ten patients during follow-up.

Eleven SELENON-RM patients (8 males, 3 females) with a median age of 20 (range 3 – 42) years were included. Nine patients were ambulant at the time of inclusion. One patient who had participated through a home visit was lost from follow-up after the first visit due to the burden of participating in this study. Patient characteristics of both cohorts can be found in Table 1.

**Table 1.** Demographic characteristics of patients with LAMA2-MD and SELENON-RM at baseline.

Demographics	LAMA2-MD		SELENON-RM	
	N	Mean ± SD	N	Mean ± SD
Age	27	21 ± 12.9 (3 - 50)	11	19.7 ± 12.9 (3 - 42)
Male (%)	9 (33%)	​	8 (73%)	​
Length (cm)	27	143.7 ± 21.0 (100.0 - 183.0)	11	147.6 ± 22.6 (97.0 – 175.0)
Weight (kg)	27	42.9 ± 16.9 (13.9 - 83.0)	11	44.1 ± 19.1 (13.2 - 70.0)
BMI (kg/m^2^) - adult	15	18.2 ± 1.0 (13.9 - 23.7)	5	22.5 ± 2.9 (19.1 - 26.3)
BMI (kg/m^2^) - child	12	21.3 ± 1.2 (13.0 - 33.9)	6	16.2 ± 3.3 (14.0 - 20.8)
Ambulant (%)	7 (26%)	​	9 (82%)	​
Respiratory support	10/27	​	8/11	​
Noninvasive ventilatory support	9/27	​	8/11	​
Invasive ventilatory support	1/27	​	0/11	​
Feeding tube^a^	7 (27)*	​	1/11	​
Facial weakness	21/27	​	11/11	​
Scoliosis OK	11 (27)	​	5 (11)	​

LAMA2-MD: LAMA2-related muscular dystrophy; SELENON-RM: SELENON-related congenital myopathy; BMI: body mass index. Data is presented mean ± SD (range) or as n (%).

^a^Increase to ten patients during follow-up.

### Clinical characteristics

#### LAMA2-MD

At baseline, 21 out of 27 LAMA2-MD patients presented with facial weakness, which remained stable during the 1.5-year follow-up period. No changes over time were observed in MRC sum score, with scores varying between 34 and 40. The most frequently and most severely affected muscles were the neck flexors and the gluteus maximus. During the follow-up period, the HHD scores increased in the following muscles: neck extensors, biceps brachii (left and right), quadriceps (left), foot plantar flexors (left) and the handgrip strength (left and right) (respectively +3.9, +1.9/+1.7, +3.7, +9.1, +2.6/+3.9, all *p*< 0.02) (Supplemental Figure 1 and Table 1). Additionally, the passive range of motion (PROM) of the elbow extension (left), hip extension (left and right), and ankle plantar flexion (right) decreased (respectively -1.3, -7.3/-9.8, -3.9, all *p*< 0.01) during the follow-up visits (Supplemental Table 1).

#### SELENON-RM

At baseline, all SELENON-RM patients presented with facial weakness, which remained unchanged during the 1.5-year follow-up period. No changes over time were observed in MRC sum score, with scores varying between 42 and 44. During the follow-up period, the HHD scores of the biceps brachii (left) and foot plantar flexors (left) increased (respectively, +1.8 and + 23.9, *p*< 0.01) (Supplemental Figure 2 and Table 2). The PROM of the wrist flexion (right), knee flexion (right) and ankle dorsal- and plantar flexion (left) decreased (respectively, -2.6, +2.3, +9.4 and -8.4, all *p*< 0.05) during the follow-up visits (Supplemental Table 2). Under our measurement convention, positive values for knee flexion and ankle dorsiflexion indicate reduced ROM.

### Functional measurements

#### MFM-20/32

**Figure 1.**
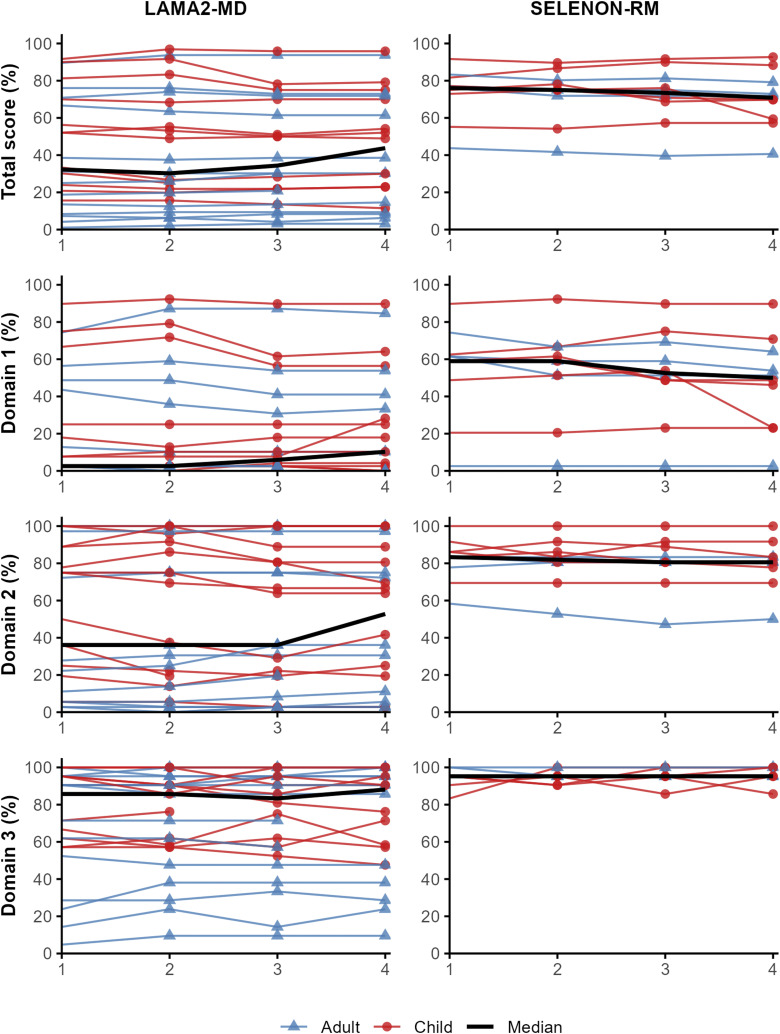
MFM-20/32 in individual LAMA2-MD and SELENON-RM patients during 1.5-year follow-up. LAMA2-MD: LAMA2-related muscular dystrophy; SELENON-RM: SELENON-related congenital myopathy; MFM-20/32: Motor function measurement-20/32.

The median MFM-20/32 total score in LAMA2-MD patients was 33.3 (18.8 – 70.0) at baseline, 30.2 (19.8 – 68.3) at 6 months, 34.4 (19.0 – 70.5) at 12 months, and 43.8 (16.7 – 71.4) at 18 months. For SELENON-RM patients, the median total scores were 76.6 (73.4 – 80.5), 75.0 (72.7 – 79.7), 73.4 (69.3 – 79.9) and, 70.8 (62.0 – 77.6) respectively. In both patient populations, domain 1 (standing and transfers) of MFM-20/32 was most severely affected, while domain 3 (distal muscle function) was relatively spared. In SELENON-RM patients, a decrease was observed in both the MFM-20/32 total score (-1.01, SE = 0.46, *p*= 0.04) and domain 1 score (-2.07, SE = 0.80, *p*= 0.02) over time. While in LAMA2-MD patients, the total MFM-20/32 score and all domains showed no changes over the 1.5 follow-up period (Tables 2 and 3, Figure 1).

**Table 2.** Clinical and functional outcome measures in LAMA2-MD patients during follow-up.

Outcome	Visit 1 (baseline)	Visit 2 (at 6 months)	Visit 3 (at 12 months)	Visit 4 (at 18 months)	P-value
​	*n (25)*	*n (25)*	*n (24)*	*n (22)*	​
**MFM-20/32 total (%)**	33.3 [18.8; 70.0]	30.2 [19.8; 68.3]	34.4 [19.0; 70.5]	43.8 [16.7; 71.4]	0.10
MFM-20/32 D1 (%)	2.6 [2.6; 43.6]	2.6 [0.0; 35.9]	5.9 [1.9; 33.3]	10.3 [0.0; 39.1]	0.26
MFM-20/32 D2 (%)	36.1 [11.1; 77.8]	36.1 [13.9; 86.1]	36.1 [16.7; 80.6]	52.8 [13.2; 79.2]	0.21
MFM-20/32 D3 (%)	90.5 [57.1; 95.2]	85.7 [57.1; 90.5]	83.3 [56.0; 95.2]	88.1 [50.0; 95.2]	0.93
​	*n (21)*	*n (23)*	*n (23)*	*n (21)*	​
**MRC sum score**	39 [23.5; 42.5]	34 [24; 41]	34 [26; 42]	40 [28; 42]	0.33
**6MWT**	*n (7)*	*n (7)*	*n (6)*	*n (7)*	​
(Distance in m)	380.0 [357.0; 465.5]	425.0 [331.0; 442.5]	446.0 [428.3; 526.0]	432.0 [368.5; 493.5]	0.07
(% Predicted)	65.1 [56.5; 75.2]	66.4 [52.6; 73.6]	71.8 [66.1; 83.1]	65.7 [57.0; 78.2]	0.14
**10-MWT**	*n (8)*	*n (8)*	*n (9)*	*n (9)*	​
Comfortable (m/s)	10.02 [9.33; 13.06]	9.52 [9.00; 12.89]	10.77 [9.30; 14.30]	10.6 [9.66; 14.68]	0.99
Maximal (m/s)	8.12 [7.08; 9.60]	7.96 [6.70; 9.51]	8.15 [6.91; 9.42]	8.23 [6.42; 9.92]	0.20
**TUG**	*n (7)*	*n (7)*	*n (7)*	*n (7)*	​
With cognitive task (s)	9.87 [9.26; 11.02]	11.92 [11.43; 11.93]	11.03 [10.38; 12.14]	10.67 [9.77; 11.00]	0.86
Without cognitive task (s)	9.7 [7.86; 10.76]	10.47 [9.44; 10.94]	10.27 [9.16; 10.79]	10.71 [8.38; 12.03]	0.08
​	*n (4)*	*n (4)*	*n (4)*	*n (4)*	​
**30s sit-to-stand (no. of stands)**	11.0 [8.8; 14.0]	12.0 [10.8; 13.8]	12.5 [11.0; 14.3]	12.0 [10.0; 14.3]	0.73
​	*n (7)*	*n (7)*	*n (7)*	*n (7)*	​
**Climb 4 stairs (s)**	3.17 [2.72; 4.22]	3.49 [2,74; 3.85]	3.23 [2.52; 3.91]	3.67 [2.47; 4.66]	0.39
​	*n (7)*	*n (7)*	*n (7)*	*n (7)*	​
**Descend 4 stairs (s)**	2.85 [2.46; 4.16]	2.95 [2.63; 3.57]	2.87 [2.05; 3.22]	2.63 [2.24; 3.76]	0.68
​	*n (8)*	*n (7)*	*n (7)*	*n (7)*	​
**Rise from floor (s)**	7.08 [5.51; 19.4]	9.00 [4.85; 19.84]	7.89 [4.81; 21.15]	7.92 [4.76; 21.22]	0.25

Several LAMA2-MD patients were unable to perform one or more specific GTFT at certain follow-up visits. Five patients had a single missed value in one of the following GTFS; rise from floor, TUG with cognitive task or 6MWT. In three cases, patients were unable to perform specific tests (climb and descend 4 stairs and rise from floor) from the baseline visit onwards were therefore excluded from the statistical analyses. LAMA2-MD: LAMA2-related muscular dystrophy; MFM-20/32: motor function measurement 20/32; MRC = Medical Research Council; 6MWT: six-minute walk test; 10-MWT: 10-meter walk test; TUG: timed up and go test. Data is presented as median [p25; p75] and n (number of patients) based on patients with valid measurements in ≥2 visits.

**Table 3.** Clinical and functional outcome measures in SELENON-RM patients during follow-up.

Outcome measure	Visit 1 (baseline)	Visit 2 (at 6 months)	Visit 3 (at 12 months)	Visit 4 (at 18 months)	P-value
​	*n (11)*	*n (10)*	*n (10)*	*n (10)*	​
**MFM-20/32 total (%)**	76.6 [73.4; 80.5]	75.0 [72.7; 79.7]	73.4 [69.3; 79.9]	70.8 [62.0; 77.6]	**0.04**
Domain 1 (%)	60.3 [51.3; 62.3]	59.0 [51.3; 65.4]	52.6 [48.7; 66.7]	50.0 [28.8; 61.5]	**0.02**
Domain 2 (%)	83.3 [79.2; 86.1]	81.9 [80.6; 85.4]	80.6 [80.6; 87.5]	80.6 [78.5; 83.3]	0.06
Domain 3 (%)	95.2 [95.2; 95.2]	95.2 [95.2; 95.2]	95.2 [95.2; 98.8]	95.2 [95.2; 100.0]	0.31
​	*n (10)*	*n (9)*	*n (8)*	*n (10)*	​
**MRC sum score**	44.0 [40.0; 46.0]	42.0 [40.0; 42.0]	42.0 [40.0; 44.0]	42.0 [40.0; 44.0]	0.38
**6MWT**	*n (8)*	*n (8)*	*n (8)*	*n (8)*	​
(Distance in m)	35.0 [274.5; 465.8]	388.0 [329.0; 440.5]	337.5 [243.0; 399.3]	332.5 [285.3; 387.0]	**0.02**
(% Predicted)	49.7 [43.3; 61.5]	52.1 [46.6; 58.6]	48.1 [37.0; 52.5]	45.9 [42.6; 53.4]	**<0.01**
**10-MWT**	*n (8)*	*n (8)*	*n (8)*	*n (8)*	​
Comfortable (m/s)	9.34 [8.78; 9.83]	9.41 [8.95; 10.21]	9.82 [9.31; 11.79]	10.50 [9.77; 12.53]	**<0.01**
Maximal (m/s)	7.38 [5.91; 8.07]	7.62 [6.35; 8.28]	7.67 [7.20; 9.54]	8.16 [6.86; 8.58]	0.08
**TUG**	*n (10)*	*n (9)*	*n (9)*	*n (8)*	​
With cognitive task (s)	12.3 [8.69; 17.46]	11.33 [9.63; 14.64]	11.30 [8.97; 12.35]	11.01 [9.07; 12.32]	0.13
Without cognitive task (s)	9.70 [8.28; 14.40]	9.48 [8.10; 10.34]	10.91 [9.10; 12.64]	9.89 [9.02; 11.21]	0.80
​	*n (4)*	*n (4)*	*n (3)*	*n (3)*	​
**30s sit-to-stand (no of stands)**	8.0 [6.0; 10.0]	8.5 [6.8; 11.3]	6.0 [5.5; 11.5]	9.5 [8.3; 11.8]	0.22
​	*n (9)*	*n (9)*	*n (9)*	*n (8)*	​
**Climb 4 stairs (s)**	3.38 [3.00; 5.72]	3.32 [2.93; 4.58]	3.94 [3.43; 4.56]	3.51 [2.88; 4.31]	0.24
​	*n (9)*	*n (9)*	*n (9)*	*n (8)*	​
**Descend 4 stairs (s)**	2.20 [2.01; 2.69]	2.23 [1.98; 2.55]	2.25 [1.91; 5.20]	2.14 [1.99; 3.38]	0.20
​	*n (8)*	*n (8)*	*n (8)*	*n (8)*	​
**Rise from floor (s)**	14.85 [8.02; 16.70]	15.80 [11.25; 23.62]	18.83 [11.77; 22.25]	17.19 [11.89; 23.66]	0.07

Two SELENON-RM patients were unable to perform specific GTFTs during follow-up: one patient was not able to perform the 30-sec sit-to-stand from visit 3 onwards; another patient was unable to perform 30-sec sit-to-stand, climb and descend 4 stairs, rise from floor and the TUG at visit 4. SELENON-RM: SELENON-related congenital myopathy; MFM-20/32: motor function measurement 20/32; MRC: Medical Research Council; 6MWT: six-minute walk test; 10-MWT: 10-meter walk test; TUG: timed up and go test. Data is presented as median [p25; p75] and n (number of patients), based on patients with valid measurements in ≥2 visits.

#### Graded timed function tests

In LAMA2-MD patients, performance on the 6MWT (distance and predicted percentage), 10MWT, TUG (with and without cognitive task), stair climbing and descending, rise from floor and 30 seconds sit-to-stand remained stable over the 1.5-year follow-up period, with no changes observed (Table 2).

In SELENON-RM patients, both walking distance (-11.18, SE = 4.43, *p*= 0.02) and percent predicted values (-1.95, SE = 0.68, *p*< 0.01) showed significant decline over time. The median distance decreased from 365 meters (% predicted: 49.7 %) at baseline to 332.5 meters (45.9%) at 1.5-year follow-up. For the 10MWT, the comfortable walking speed (0.54, SE = 0.17, *p*< 0.01) showed a significant decrease over time. No changes were observed in TUG (with and without cognitive task), stair climbing and descending, rise from floor and 30 seconds sit-to-stand over time (Table 3).

### Accelerometry

Twenty-four LAMA2-MD and all^11^ SELENON-RM patients wore the GeneActiv device during follow-up. For LAMA2-MD patients, the percentage of moderate activity during the 7-day period increased (0.08, SE = 0.03, *p*< 0.01) over time, while other outcome variables showed no changes. For SELENON-RM patients, no significant changes were observed (Table 4).

**Table 4.** Accelerometry in LAMA2-MD and SELENON-RM patients during follow-up.

​	Visit 1 (baseline)	Visit 2 (6 months)	Visit 3 (12 months)	Visit 4 (18 months)	P-value
**LAMA2-MD**	*n (24)*	*n (24)*	*n (23)*	*n (22)*	​
% Sedentary activity per 24 h	95.2 [90.7; 99.3]	95.5 [91.2; 99.1]	95.1 [91.3; 99.0]	95.5 [88.9; 99.0]	0.49
% Light activity per 24 h	2.8 [0.6; 7.1]	2.7 [0.6; 6.9]	3.0 [0.7; 5.9]	2.8 [0.7; 7.4]	0.58
% Moderate activity per 24 h	1.3 [0.1; 2.4]	1.2 [0.1; 2.8]	1.5 [0.1; 2.2]	1.3 [0.2; 3.3]	**< 0.01**
% Vigorous activity per 24 h	0.0 [0.0; 0.2]	0.1 [0.0; 0.2]	0.1 [0.0; 0.3]	0.1 [0.0; 0.2]	0.46
No. counts per day	189364 [110931; 251252]	182540 [113606; 263409]	208089 [94176; 238508]	202868 [120529; 273714]	0.25
**SELENON-RM**	*n (11)*	*n (11)*	*n (11)*	*n (11)*	​
% Sedentary activity per 24 h	91.3 [90.3; 93.9]	91.0 [87.3; 93.9]	91.0 [89.6; 93.4]	92.5 [91.5; 93.6]	0.86
% Light activity per 24 h	4.6 [4.1; 6.5]	4.3 [3.3; 7.4]	4.6 [3.8; 7.2]	3.8 [3.2; 6.6]	0.60
% Moderate activity per 24 h	2.2 [1.1; 3.6]	2.2 [1.5; 4.1]	1.7 [1.6; 3.5]	2.3 [1.5; 2.9]	0.95
% Vigorous activity per 24 h	0.6 [0.1; 1.2]	0.7 [0.2; 1.0]	0.5 [0.1; 1.2]	0.7 [0.2; 1.0]	0.41
No. counts per day	268934 [228489; 289800]	278392 [225308; 291352]	263879 [224774; 293783]	233447 [215284; 310576]	0.21

LAMA2-MD: LAMA2-related muscular dystrophy; SELENON-RM: SELENON-related congenital myopathy; No. counts per day: number of counts per day. Data is presented as median [p25; p75] and n (amount of patients).

### Questionnaires

In the PedsQL Generic Core questionnaire, an increase (4.1, SE = 1.8, p = 0.03) in the social functioning domain score was observed in the children’s proxy of the LAMA2-MD patients, however, this change was not observed in the parents’ proxy of the LAMA2-MD and nor in the proxies of the SELENON-RM patients (Tables 5 and 6 and Supplemental Tables 3 and 4). No changes were found over time in the other domains or in the total scores both the children’s and parents’ proxies in either group. An increase (2.3, SE = 1.0, p = 0.03) on the sleep/rest fatigue domain of the PedsQL MFS was observed in the children’s proxy of the LAMA2-MD patients, while this change was not observed in the parents’ proxy and the SELENON-RM patients (Tables 5 and 6 and Supplemental Tables 3 and 4). No other domains or the total score demonstrated changes in either group. Further, no changes in domain or total score were observed in both groups over time on the PedsQL NMM. Analysis of the RAND-36 showed an increase (3.9, SE = 1.5, p = 0.02), indicating an increase in quality of life, in the domain of role limitations due to emotional problems in LAMA2-MD patients (Table 5 and supplement Tables 3 and 4). In SELENON-RM patients, a decrease (-6.3, SE = 2.5, p = 0.03) on the social functioning domain score was found (Table 6 and Supplemental Tables 3 and 4). Other domains and total score showed no changes for both groups during the follow-up period. In the INQoL questionnaire, the weakness domain score decreased (-3.6, SE = 1.4, p < 0.01) in LAMA2-MD patients over time, while all other domains remained unchanged (Table 5). No changes were observed in any domains in SELENON-RM patients. In SELENON-RM patients the CIS-fatigue subscore decreased (-1.3, SE = 0.5, *p*= 0.01) over time (Supplemental Tables 3 and 4). In LAMA2-MD patients, no changes were observed during follow-up.

**Table 5.** Questionnaires in LAMA2-MD patients during follow-up.

Questionnaire	Visit 1 (baseline)	Visit 2 (6 months)	Visit 3 (12 months)	Visit 4 (18 months)	P-value
**PedsQL Generic**	*n (12)*	*n (12)*	*n (11)*	*n (11)*	​
Parents proxy	59.0 (±13.1)	57.7 (±11.3)	56.5 (±12.0)	60.5 (±10.2)	0.82
Childrens proxy	62.4 (±7.1)	64.0 (±7.3)	65.0 (±9.4)	63.2 (±12.9)	0.77
**PedsQL NMM**	​	​	​	​	​
Parents proxy	61.8 (±14.9)	65.1 (±10.3)	64.2 (±12.7)	65.7 (±9.5)	0.06
Childrens proxy	65.8 (±12.4)	67.2 (±8.9)	67.0 (±11.6)	67.7 (±9.6)	0.16
**PedsQL MFS**	​	​	​	​	​
Parents proxy	76.4 (±13.3)	76.4 (±10.9)	78.1 (±8.9)	77.7 (±9.6)	0.07
Childrens proxy	76.1 (±9.6)	76.6 (±8.8)	78.0 (±10.4)	79.9 (±8.8)	0.11
**RAND-36**	*n (14)*	*n (14)*	*n (14)*	*n (13)*	​
Physical functioning	15.00 (±25.9)	13.6 (±23.7)	15.0 (±25.7)	16.5 (±27.1)	1.00
Bodily pain	89.5 (±13.3)	85.7 (±18.3)	87.1 (±16.1)	84.4 (±15.9)	0.10
Role limitations; physical health	73.2 (±37.3)	73.2 (±30.2)	76.8 (±34.6)	67.3 (±35.9)	0.55
Role limitations; emotional health	88.1 (±28.1)	95.2 (±17.8)	95.2 (±17.8)	100.0 (±0.0)	**0.02**
Emotional well-being	87.4 (±9.1)	88.6 (±12.8)	88.6 (±10.2)	85.9 (±10.4)	0.53
Social functioning	84.8 (±22.0)	80.4 (±24.9)	79.5 (±21.7)	82.7 (±20.1)	0.59
Energy/Fatigue	67.5 (±17.6)	70.4 (±15.9)	73.9 (±14.4)	70.8 (±16.1)	0.53
General health perceptions	45.7 (±27.7)	47.9 (±22.8)	51.1 (±19.0)	49.2 (±25.2)	0.38
**INQoL**	*n (15)*	*n (14)*	*n (14)*	*n (13)*	​
Weakness	60.4 (±22.1)	61.3 (±25.6)	50.4 (±29.0)	52.2 (±28.1)	**< 0.01**
Muscle locking	6.3 (±18.5)	2.6 (±9.9)	1.1 (±4.2)	0.8 (±2.9)	0.11
Pain	9.8 (±18.5)	10.9 (±18.6)	11.7 (±14.9)	9.7 (±13.0)	0.52
Fatigue	17.2 (±19.3)	20.3 (±25.9)	16.9 (±20.1)	18.6 (±19.3)	0.79
Activities	47.0 (±30.5)	46.4 (±25.5)	43.9 (±22.8)	46.2 (±24.3)	0.999
Independence	62.9 (±31.2)	61.6 (±30.0)	55.6 (±26.7)	57.6 (±25.5)	0.45
Social relationships	18.8 (±22.9)	12.5 (±17.6)	10.8 (±15.8)	14.3 (±17.1)	0.09
Emotions	11.3 (±14.1)	11.9 (±22.2)	4.2 (±8.6)	7.1 (±8.9)	0.09
Body image	36.3 (±35.8)	29.6 (±31.8)	29.2 (±28.9)	25.4 (±25.4)	0.29
​	*n (15)*	*n (14)*	*n (14)*	*n (13)*	​
**CIS**	79.6 (±8.6)	82.9 (±7.6)	79.6 (±5.8)	79.5 (±8.2)	0.70
**IPA**	*n (15)*	*n (14)*	*n (14)*	*n (13)*	​
Autonomy indoors	5.3 (±4.7)	5.1 (±3.5)	5.6 (±3.4)	5.8 (±3.4)	0.19
Autonomy outdoors	6.1 (±3.3)	5.6 (±2.4)	6.3 (±3.2)	6.8 (±4.0)	0.33
Family role	3.1 (±2.1)	3.4 (±2.8)	4.1 (±2.4)	3.8 (±2.4)	**0.03**
Social	5.2 (±2.1)	5.4 (±3.8)	5.0 (±2.8)	6.0 (±3.5)	0.16
Work/Education	10.3 (±4.7)	11.9 (±4.9)	11.0 (±5.7)	10.2 (±4.3)	0.78
**ACTIVLIM**	*n (25)*	*n (24)*	*n (24)*	*n (23)*	​
Easy	4.7 (±5.6)	4.8 (±5.5)	4.2 (±5.4)	4.7 (±5.6)	0.10
Difficult	2.2 (±2.4)	2.4 (±2.4)	2.5 (±2.6)	2.4 (±2.3)	0.80
Impossible	11.0 (±7.5)	10.6 (±7.3)	11.3 (±7.1)	10.9 (±7.0)	**0.03**

LAMA2-MD: LAMA2-related muscular dystrophy; PedsQL: Pediatric quality of life inventory; NMM: Neuromuscular Module; MFS: Multidimensional Fatigue Scale; RAND-36: Research and development-36; INQoL: Individualized neuromuscular quality of life; CIS: Checklist individual strength; IPA: Impact on participation and autonomy; ACTIVLIM: Activity limitations questionnaire. Data is presented as mean ± SD.

**Table 6.** Questionnaires SELENON-RM patients during follow-up.

Questionnaire/domain	Visit 1 (baseline)	Visit 2 (6 months)	Visit 3 (12 months)	Visit 4 (18 months)	P-value
**PedsQL Generic**	*n (6)*	*n (6)*	*n (6)*	*n (5)*	​
Parents proxy	52.7 (±8.5)	55.0 (±8.8)	57.7 (±6.8)	50.6 (±5.7)	0.82
Childrens proxy	55.7 (±15.1)	63.7 (±21.4)	56.3 (±14.0)	51.1 (±15.6)	0.77
**PedsQL NMM**	​	​	​	​	​
Parents proxy	58.5 (±13.1)	64.3 (±14.1)	65.8 (±18.1)	60.6 (±9.6)	0.06
Childrens proxy	60.5 (±16.0)	65.4 (±14.6)	67.1 (±17.8)	63.1 (±9.7)	0.16
**PedsQL MFS**	​	​	​	​	​
Parents proxy	58.5 (±13.1)	64.3 (±14.1)	65.8 (±18.1)	60.6 (±9.6)	0.07
Childrens proxy	60.5 (±16.0)	65.4 (±14.6)	67.1 (±17.8)	63.1 (±9.7)	0.11
**RAND36**	*n (3)*	*n (4)*	*n (5)*	*n (5)*	​
Physical functioning	13.3 (±14.4)	27.5 (±21.0)	24.0 (±18.2)	19.0 (±15.6)	0.26
Bodily pain	79.2 (±27.9)	65.0 (±19.0)	77.0 (±19.2)	74.5 (±18.9)	0.82
Role limitations; physical health	66.7 (±38.2)	37.5 (±43.3)	50.0 (±35.4)	40.0 (±37.9)	0.22
Role limitations; emotional health	100.0 (±0.0)	91.7 (±16.7)	100.0 (±0.0)	100.0 (±0.0)	0.48
Emotional well-being	74.7 (±12.9)	82.0 (±10.6)	80.8 (±14.8)	76.8 (±13.7)	0.64
Social functioning	70.8 (±28.9)	68.8 (±12.5)	57.5 (±24.4)	57.5 (±20.9)	**0.03**
Energy/Fatigue	45.0 (±5.0)	46.3 (±2.5)	49.0 (±8.2)	46.0 (±12.9)	0.97
General health perceptions	43.3 (±20.2)	50.0 (±23.5)	24.0 (±12.9)	30.0 (±10.6)	**0.05**
**INQoL**	*n (5)*	*n (5)*	*n (5)*	*n (5)*	​
Weakness	74.7 (±8.7)	74.7 (±10.1)	68.4 (±13.4)	73.7 (±8.3)	0.39
Muscle locking	4.2 (±9.4)	6.3 (±14.1)	4.2 (±9.4)	4.2 (±9.4)	0.67
Pain	36.8 (±35.3)	40.0 (±41.2)	32.6 (±38.2)	36.8 (±24.4)	0.74
Fatigue	51.6 (±20.5)	45.3 (±17.3)	53.7 (±20.2)	57.9 (±17.5)	0.07
Activities	55.9 (±19.0)	49.1 (±23.2)	51.3 (±22.6)	50.7 (±21.9)	0.27
Independence	41.0 (±29.1)	45.3 (±33.5)	46.0 (±28.5)	47.1 (±31.1)	0.18
Social relationships	14.8 (±14.2)	19.6 (±20.9)	15.9 (±20.2)	22.2 (±18.5)	0.15
Emotions	29.4 (±15.3)	30.6 (±10.2)	32.8 (±12.2)	26.7 (±18.7)	0.73
Body image	57.2 (±20.1)	67.2 (±9.5)	57.8 (±25.0)	62.2 (±12.8)	0.85
​	*n (5)*	*n (5)*	*n (5)*	*n (5)*	​
**CIS**	85.6 (±3.6)	82.2 (±5.0)	82.8 (±5.6)	80.8 (±1.3)	**0.04**
**IPA**	*n (5)*	*n (5)*	*n (5)*	*n (5)*	​
Autonomy indoors	3.8 (±3.1)	4.4 (±3.4)	3.2 (±3.1)	3.6 (±4.5)	0.71
Autonomy outdoors	9.4 (±3.3)	10.2 (±4.5)	10.0 (±4.5)	9.2 (±4.8)	0.76
Family role	4.4 (±3.2)	4.8 (±1.8)	3.2 (±2.6)	4.2 (±3.6)	0.49
Social	4.0 (±2.6)	5.2 (±2.8)	6.2 (±3.6)	6.0 (±2.7)	**<0.01**
Work/Education	8.0 (±3.2)	7.2 (±4.4)	6.6 (±4.0)	7.0 (±4.1)	0.52
**ACTIVLIM**	*n* (10)	*n* (10)	*n* (10)	*n* (9)	​
Easy	9.9 (±5.0)	8.9 (±5.1)	9.0 (±5.8)	8.1 (±6.4)	**0.04**
Difficult	5.4 (±3.8)	6.5 (±4.1)	5.2 (±4.2)	6.0 (±5.2)	0.99
Impossible	2.6 (±3.5)	2.6 (±3.5)	3.6 (±4.4)	3.9 (±4.1)	**<0.01**

SELENON-RM: SELENON-related congenital myopathy; PedsQL: Pediatric quality of life inventory; NMM: Neuromuscular Module; MFS: Multidimensional Fatigue Scale; RAND-36: Research and development-36; INQoL: Individualized neuromuscular quality of life; CIS: Checklist individual strength; IPA: Impact on participation and autonomy; ACTIVLIM: Activity limitations questionnaire. Data is presented as mean ± SD.

For the IPA questionnaire, the family role domain score increased in LAMA2-MD patients during follow-up (0.3, SE = 0.1, *p*= 0.03) (Table 5). In SELENON-RM patients, the social functioning domain score increased during follow-up (0.7, SE = 0.2, *p*< 0.01) (Table 6). No other domain scores showed changes during follow-up.

In the ACTIVLIM, an increase in tasks scored as impossible (0.25, SE = 0.12, p = 0.04) was observed over time in LAMA2-MD patients (Table 5). For SELENON-RM patients, an increase in tasks scored as impossible (0.45, SE = 0.15, *p*< 0.01) and a decrease in tasks scored as easy (-0.45, SE = 0.20, *p*= 0.04) was observed over time (Table 6).

On the EK2, McGill pain questionnaire and Wong-Baker faces pain rating scale no changes were observed during the follow-up period in both the LAMA2-MD and SELENON-RM patients (Supplemental Tables 3 and 4).

## Discussion

This 1.5-year follow-up study in patients with LAMA2-MD and SELENON-RM showed clinically minimal overall changes, indicating predominantly stable motor function over this timeframe. This stability aligns with the natural history observed in other slowly progressive congenital muscle diseases with an early onset.^46^ While this stability may be reassuring from a clinical management perspective, it poses challenges for the design and execution of clinical trials, which will be discussed below.

Our study provides insights into disease severity and progression in LAMA2-MD and SELENON-RM patients. Although baseline findings showed that MFM-20/32 total and domain 2 scores were suitable and feasible for measuring disease severity in LAMA2-MD patients, the 1.5-year longitudinal data showed no significant changes over time in these scores. Similarly, other functional outcome measures showed no significant changes over this period. These findings suggest that the currently investigated outcome measures lack the sensitivity to capture disease progression within a 1.5-year time period with subsequent visits every 6 months. Our findings are consistent with those observed in other slowly progressive congenital muscle diseases, where clinical and functional outcome measures often remained stable over a 1.5-year follow-up period.^46,47^ But in contrast with more rapid progressive neuromuscular disorders such as DMD and spinal muscular atrophy.^22,29^ Given that LAMA2-MD is a slowly progressive congenital muscle disease, it is plausible that the rate of functional decline is too small to be detected using these outcome measures within the 1.5-year follow-up. This has important implications for future clinical trials with a typical duration of 1 to 2 years, where these outcome measures may similarly fail to capture meaningful progression, highlighting the need for more sensitive clinical endpoints or for biomarkers that are used as surrogate endpoints, such as imaging techniques.

Baseline findings suggested that the MFM-20/32, 6MWT and the ‘rise from floor’ graded timed function test may be valuable outcome measures for assessing disease severity and progression in SELENON-RM patients. Our 1.5-year follow-up data shows that both the MFM-20/32 total and domain 1 score, as well as the 6MWT, demonstrated significant changes over time. These findings suggest that the investigated outcome measures may be sensitive to capture disease progression in SELENON-RM patients and could serve as a potential outcome measures in future clinical trials. However, it is important to interpret these results with caution, as the observed changes are small and obtained from a limited sample size. In contrast, the ‘rise from floor’ did not show significant changes, suggesting it may be less sensitive for monitoring progression over 1.5-year in SELENON-RM patients. This contrasts with findings in DMD, where this test has been shown to be a relevant outcome measure for monitoring and predicting disease progression.^22^ A possible explanation for this is that DMD is a more rapid progressive neuromuscular disorder, allowing functional decline to be captured over shorter time intervals.

Interestingly, the HHD results in LAMA2-MD patients showed an increase over time, which was unexpected given that muscle strength typically declines in progressive congenital muscle diseases. The most likely explanation for this finding is that the LAMA2-MD cohort includes 12 patients under 18 years of age, of whom 8 are 12 years or younger. As shown in Supplemental Figure 1, increases in the HHD scores over time were predominantly observed in the children, whereas adult patients showed stable or declining values. Since muscle strength naturally increases during childhood and adolescence, albeit at a reduced rate compared to healthy peers, this developmental gain may account for the observed increase in HHD values over time.^48^ These findings underscore how physiological growth can affect not only clinical and functional outcome measures, but potentially also other outcome measures (e.g. imaging). In pediatric patients, normal development may at times progress more rapidly than the underlying disease, which presents challenges in the interpretation of longitudinal changes. Additionally, the observed differences between left and right-side measurements may be attributed to contractures that limit proper assessment. Furthermore, since HHD provides a more precise strength measurement compared to the MRC scale, these side-to-side differences were not detected using the MRC scale.

Other functional outcome measures (Hammersmith Functional Motor Scale (HFMS), Pediatric Balance Scale (PBS), Mini Balance Evaluation Systems Test (miniBEST)) that were investigated in the LAST STRONG study were considered neither suitable nor feasible for assessing disease severity at baseline.^20,21^ This was due to pronounced floor effects in non-ambulant patients for the HFMS, and limited ability to perform the required tasks for the miniBEST and PBS. Therefore, these measures were excluded from 1.5 year follow-up analyses.

Given the limited sensitivity of functional outcome measures in LAMA2-MD and SELENON-RM over a 1.5-year follow-up, alternative approaches may be necessary to detect subtle changes.^17–19^ One of those might be muscle imaging with, for instance MRI and/or muscle ultrasound. In facioscapulohumeral muscular dystrophy, muscle MRI and muscle ultrasound have already demonstrated potential as a biomarkers suggesting they may also serve as possible biomarkers in LAMA2-MD and SELENON-RM as well.^49^ In the LAST STRONG study, muscle imaging techniques were included but these imaging data have yet to be analyzed and will be published in the near future.

The questionnaires used in our study provide valuable insights into patient experiences on quality of life, pain, fatigue, and activities and participation. However, they offer limited information regarding the functional progression over the 1.5-year follow-up. To better capture subtle changes over time, it is important to consider alternative patient-reported outcome measures that are more sensitive to change. Rasch-built questionnaires have demonstrated an increased sensitivity to change in other childhood-onset muscle diseases.^50^ However, such instruments are currently not available for LAMA2-MD and SELENON-RM.

For accelerometry, we observed a significant increase over time in the percentage of moderate activity in LAMA2-MD patients. Baseline data from both cohorts demonstrated strong correlations between accelerometry and MFM-20/32 scores, including associations with sedentary, light, and moderate activity levels.^20,21^ Notably, in our longitudinal analysis, MFM-20/32 total and domain scores remained stable in LAMA2-MD patients, suggesting that accelerometry devices may capture subtle changes in activity patterns that conventional functional outcome measures fail to detect. In contrast, SELENON-RM patients showed no significant changes in accelerometry over time, whereas MFM-20/32 total and domain 1 score showed a small but significant decline. These contrasting findings highlight potential differences in the sensitivity of accelerometry versus conventional outcome measures across disease groups and underscore the need for further longitudinal validation to establish whether accelerometry can be used as a reliable clinical trial endpoint.

Major strengths of our cross-sectional longitudinal study are inclusion of a wide age range and broad spectrum of disease severity, as well as a comprehensive set of clinical and functional outcome measures. Further, the study cohort includes of the large majority of the LAMA2-MD and SELENON-RM patients identified in the Netherlands and Flanders. This results in a high participation rate that minimizes selection bias and strengthens the generalizability of our findings. Building on previously published 1.5-year follow-up data on bone quality, respiratory and cardiac outcomes, this manuscript further expands the knowledge on LAMA2-MD and SELENON-RM.^17,19^ Furthermore, the basket design enables efficient data collection on two ultra-rare diseases, and therefore, provides information on disease progression in both pediatric and adult populations.

An important limitation of this study is that analyses were not stratified by age or ambulatory status. Although such stratification could provide additional insights and is highly relevant for future clinical trial design, this was not feasible due to the limited number of participants in each subgroup which would have resulted in underpowered analyses and unreliable results.

Another limitation is that several patients were not able to perform the GTFTs during all follow-up visits. In most cases, this was due to clinical deterioration that resulted in being unable to perform the task. While this suggests disease progression, such cases were recorded as a missing data point and subsequently excluded from data analysis if this was the case at more than 2 visits. This may have contributed to an underestimation of disease progression in these outcome measures, potentially masking meaningful functional decline in these individuals. The inability to perform the task as a result of functional decline reflects meaningful disease progression and shows how these outcome measures may fail to fully capture the natural course of the disease, particularly in more severely affected patients.

Lastly, for the MRC sum score, if patients had contractures in one of the joints, they were excluded from analysis. This decision was made to maintain consistency across assessments, but may have led to an underestimation of the MRC sum score, especially if the contracture rather than the muscle weakness itself was the limiting factor. Contractures are common in both LAMA2-MD and SELENON-RM patients and may interfere with the reliability and validity of clinical and functional outcome measures. Since most of those measures, including MRC and HHD, do not account for limitations in range of motion on performance, those outcome measures may not be suitable for accurately assessing disease progression in these patient cohorts.

In conclusion, LAMA2-MD patients remained largely stable and SELENON-RM patients showed minimal overall progression over the 1.5-year follow-up period. In LAMA2-MD patients, the investigated clinical and functional outcome measures do not seem to be suitable to capture disease progression within this follow-up period. In SELENON-RM, both the MFM-20/32 total and domain 1 score and the 6MWT demonstrated declines over time. However, these changes were small and the suitability of these outcome measures is limited to ambulant patients, reducing their feasibility in more severely affected individuals. Motor development continues throughout childhood and occurs in parallel with disease progression. Thus, developmental gain may influence outcome measures, as motor development may progress faster than functional decline, and complicate interpretation. As our findings reflect the entire age spectrum, future clinical trials will likely require separate designs for pediatric and adult populations to account for developmental gains.

Combined with previously published data of this cohort on respiratory, cardiac, and bone health data, these findings contribute to a growing toolbox of outcome measures for LAMA2-MD and SELENON-RM. This integrated approach will be essential for designing clinical trials and selecting sensitive outcome measures. Imaging outcomes from the LAST STRONG study are expected to further strengthen this toolbox.

Further research is needed to optimize and select relevant and sensitive serum biomarkers, imaging biomarkers, and clinical outcome measures to evaluate disease progression in LAMA2-MD and SELENON-RM. Given the limited sensitivity of several current investigated outcome measures over a 1.5-year time period, future studies should prioritize the development and validation of disease-specific outcome measures with improved responsiveness to subtle functional changes. As LAMA2-MD and SELENON-RM are slowly progressive congenital muscle diseases, longer-term follow-up studies are necessary to identify consistent and clinically significant declines in function. A 5-year follow-up study is currently ongoing, as well as efforts to standardize longitudinal data collection as part of routine clinical care. Collaboration with clinicians, researchers and members of the biotech industry preparing for phase 1 and 2 trials are already underway during monthly meetings and are an important step toward trial readiness. Importantly, both congenital muscle diseases show distinct natural histories, and underscore the need for tailored trial designs. Age- and phenotype-stratified analyses will be essential to inform clinical trial design and ensure that therapeutic approaches are tailored. Larger international cohorts, facilitated through data sharing, will be needed to enable subgroup analyses and capture the full clinical spectrum.

## Supplemental material


Supplemental Material - Clinical and functional outcome measures in LAMA2-related muscular dystrophy and SELENON-related myopathy; a 1.5-year natural history study
Supplemental Material for Clinical and functional outcome measures in LAMA2-related muscular dystrophy and SELENON-related myopathy; a 1.5-year natural history study by Elisabeth C. M. de Laat, Jan T. Groothuis, Karlijn Bouman, Saskia L. S. Houwen – van Opstal Corrie E. Erasmus, Nicol C. Voermans in Journal of Neuromuscular Diseases.

## Data Availability

The data that support the findings of this study are available from the corresponding author on reasonable request.*

## Appendix

### Glossary

6MWT: 6-Minute Walk test
10MWT: 10-meter walk test
ACTIVLIM: Activity limitations questionnaire
CIS: Checklist Individual Strength
DMD: Duchenne muscular dystrophy
EK2: Egen Klassification version 2
HFMS: Hammersmith Functional Motor Scale
HHD: Hand-held dynamometry
INQoL: Individualized Neuromuscular Quality of Life
IPA: Impact on Participation and Autonomy
GTFTs: Graded and timed function tests
LAMA2-MD: Laminin-a2-related muscular dystrophy
MFM-20/32: Motor function measure – 20/32
MiniBEST: Mini Balance Evaluation Systems Test
MRI: Magnetic resonance imaging
MRC: Medical Research Council
PBS: Pediatric Balance Scale
PedsQL: Pediatric Quality of Life Inventory
PROM: Passive range of motion
RAND-36: Research and Development-36
SELENON-RM: SELENON-related myopathy
SVMgs: The gravity of subtracted sum of vector magnitudes
TUG: Timed up and go test
