# Supplementary material for: Clinical and functional outcome measures in LAMA2-related muscular dystrophy and SELENON-related myopathy; a 1.5-year natural history study
Source: J Neuromuscul Dis. 2026 Aug 1:22143602261465467. Online ahead of print. doi: 10.1177/22143602261465467 (PMC13438859; doi:10.1177/22143602261465467)
Supplement: Supplementary file 1 [file sj-pdf-1-jnd-10.1177_22143602261465467.pdf]

Supplementary tables

Supplementary table 1. LAMA2-MD clinical features during follow-up.

	Visit 1 (baseline)	Visit 2 (at 6 months)	Visit 3 (at 12 months)	Visit 4 (at 18 months)	P-value
**HHD (Newton)**					
Neck extensor	64.0 [33.2 ;79.7]	67.7 [34.7 ;91.0]	75.2 [51.3 ;94.6]	90.3 [69.3 ;111.7]	**< 0.01**
Biceps brachii					
- *Right*	34.7 [20.7 ;60.0]	33.8 [17.3 ;65.8]	39.3 [20.3 ;70.0]	43.0 [24.7 ;73.3]	**0.02**
- *Left*	37.3 [18.7 ;60.0]	35.7 [17.8 ;64.8]	39.7 [20.0 ;67.0]	46.2 [23.6 ;72.3]	**< 0.01**
Triceps brachii					
- *Right*	29.0 [21.5 ;65.8]	40.0 [208 ;67.5]	31.3 [19.7 ;59.7]	29.3 [19.2 ;61.5]	0.85
- *Left*	29.3 [18.8 ;53.8]	41.5 [16.8 ;64.2]	34.0 [18.7 ;53.7]	34.3 [17.2 ;53.7]	0.95
Handgrip strength					
- *Right*	24.7 [16.3 ;36.7]	34.5 [21.9 ;68.8]	32.5 [19.5 ;68.0	35.5 [21.5 ;59.9]	**< 0.01**
- *Left*	22.0 [17.3 ;45.3]	40.3 [20.8 ;66.3]	32.7 [20.9 ;65.5]	36.0 [22.5 ;54.7]	**0.01**
Quadriceps					
- *Right*	84.5 [59.7 ;127.8	84.0 [47.8 ;124.2]	84.8 [59.3 ;128.3]	79.0 [53.4 ;133.3]	0.27
- *Left*	99.3 [45.7 ;128.4]	104.0 [45.0 ;122.0]	100.8 [65.0 ;130.8]	95.7 [52.3 ;144.8]	**< 0.01**
Foot extensors					
- *Right*	62.0 [43.7 ;116.0]	82.5 [54.4 ;124.0]	78.7 [44.2 ;117.7]	69.83 [44.7 ;111.9]	0.81
- *Left*	50.3 [26.5 ;109.2]	79.0 [43.8 ;111.5	74.3 [40.0 ;106.0]	63.7 [43.7 ;116.0]	0.40
Foot plantar flexors					
- *Right*	n.a[a]	n.a[a]	n.a[a]	n.a[a]	n.a[a]
- *Left*	92.7 [39.5 ;120.4]	97.3 [40.8 ;215.0]	101.2 [36.3 ;162.4]	93.3 [42.3 ;225.7	**0.02**
**PROM (degrees)**					
Wrist flexion					
- *Right*	92.5 [81.3 ;100.0]	90.0 [85.0 ;110.0]	85.0 [75.0 ;95.0]	85.0 [80.0 ;97.5]	0.70
- *Left*	97.5 [90.0 ; 118.8]	90.0 [85.0 ;120.0]	90.0 [85.0 ;120.0]	90.0 [85.0 ;112.5]	0.73
Wrist extension					
- *Right*	100.0 [86.3 ;136.3]	95.0 [85.0 ;121.3]	100.0 [95.0 ;170.0]	100.0 [87.5 ;127.5]	0.89
- *Left*	105.0 [90.0 ;138.8]	105.0 [90.0 ;137.5]	105.0 [90.0 ;160.0]	105.0 [85.0 ;152.5]	0.56

Elbow flexion					
- *Right*	35.0 [30.0 ;48.8]	40.0 [30.0 ;50.0]	40.0 [30.0 50.0]	40.0 [35.0 47.5]	0.17
- *Left*	35.0 [30.0 ;49.5]	40.0 [30.0 ;48.8]	35.0 [30.0 ;50.0]	40.0 [30.0 ;50.0]	0.69
Elbow extension					
- *Right*	147.5 [102.5 ;170.0]	140.0 [101.3 ;165.0]	150.0 [100.0 ;170.0]	150.0 [107.5 ;167.5]	0.10
- *Left*	142.5 [110.0 ;168.8]	142.5 [101.3 ;168.8]	150.0 [100.0 ;165.0]	150.0 [115.0 ;167.5]	**< 0.01**
Hip flexion					
- *Right*	65.0 [46.3 ;80.0]	70.0 [40.0 ;80.0]	60.0 [40.0 ;80.0]	60.0 [42.5 ;92.5]	0.56
- *Left*	70.0 [50.0 ;80.0]	62.5 [46.3 ;80.0]	60.0 [40.0 ;75.0]	60.0 [40.0 ;80.0]	0.09
Hip extension					
- *Right*	130.0 [45.0 ;47.5]	130.0 [15.0 ;142.5]	120.0 [15.0 ;130.0]	110.0 [15.0 ;130.0]	**< 0.01**
- *Left*	132.5 [101.3 ;150.0]	125.0 [15.0 ;131.3]	115.0 [15.0 ;130.0]	110.0 [15.0 ;135.0]	**< 0.01**
Knee flexion					
- *Right*	50.0 [35.0 ;65.0]	52.5 [35.0 ;70.0]	50.0 [40.0 ;70.0]	55.0 [40.0 ;70.0]	0.20
- *Left*	50.0 [40.0 ;70.0]	55.0 [35.0 ;70.0]	55.0 [40.0 ;70.0]	55.0 [37.5 ;70.0]	0.81
Knee extension					
- *Right*	157.5 [121.3 ;175.0]	162.5 [135.0 ;175.0]	160.0 [120.0 ;180.0]	160.0 [130 ;175.0]	0.13
- *Left*	157.5 [120.0 ;175.0]	160.0 [126.3 ;175.0]	160.0 [120.0 ;180.0]	155.0 [122.5 ;175.0]	0.53
Ankle flexion					
- *Right*	137.5 [115.0 ;157.5]	140.0 [115.0 ;155.0]	135.0 [130.0 ;160.0]	150.0 [130.0 ;160.0]	**< 0.01**
- *Left*	140.0 [102.5 ;155.0]	137.5 [116.3 ;155.0]	140.0 [120.0 ;160.0]	150.0 [130.0 ;160.0]	0.10
Ankle extension					
- *Right*	117.5 [101.3 ;130.0]	105.0 [95.0 ;130.0]	100.0 [90.0 ;130.0]	95.0 [90.0 ;122.5]	**<0.01**
- *Left*	112.5 [95.0 ;130.0]	120.0 [95.0 ;133.8]	100.0 [90.0 ;135.0]	100.0 [90.0 ;130.0]	0.56

[a] *HHD plantar flexors right: excluded from mixed model since no participants were able to complete this measurement ≥ 2 visits.*

LAMA2-MD = LAMA2-related muscular dystrophy; HHD = Hand-held dynamometry. Data is presented as median [p25 ; p75].

Supplementary table 2. SELENON-RM clinical features during follow-up

	Visit 1 (baseline)	Visit 2 (at 6 months)	Visit 3 (at 12 months)	Visit 4 (at 18 months)	P-value
HHD (Newton)					
Neck extensor	79.0 [67.9 ;82.6]	80.8 [67.1 ;92.3]	70.0 [63.3 ;83.7]	74.3 [67.7 ;93.5]	0.72
Biceps brachii					
- *Right*	55.0 [54.7 ;60.0]	55.7 [52.7 ;61.0]	55.0 [54.0 ;63.7]	57.0 [56.0 ;65.3]	**0.05**
- *Left*	54.7 [52.3 ;65.3]	58.0 [51.7 ;63.7]	62.8 [55.5 ;74.1]	59.7 [55.7 ;65.7]	**< 0.01**
Triceps brachii					
- *Right*	35.7 [31.7 ;52.0]	38.0 [32.3 ;52.7]	44.7 [33.3 ;52.0]	38.7 [32.0 ;51.7]	0.54
- *Left*	35.7 [32.7 ;44.0]	36.3 [32.3 ;46.3]	38.7 [28.3 ;52.8]	39.7 [34.0 ;49.0]	0.20
Handgrip strength					
- *Right*	36.0 [31.0 ;52.0]	39.0 [28.7 ;60.0]	51.7 [33.3 ;71.3]	41.3 [34.0 ;49.7]	0.31
- *Left*	32.3 [29.3 ;45.0]	41.3 [35.7 ;63.0]	53.7 [33.0 ;70.0]	43.3 [29.3 ;46.3]	0.49
Quadriceps					
- *Right*	111.0 [68.3 ;117.0]	108.0 [103.3 ;123.7]	113.7 [85.3 ;121.3]	120.8 [99.8 ;146.7]	0.22
- *Left*	118.3 [76.7 ;129.0]	121.0 [90.7 ;126.3]	106.3 [85.3 ;121.0]	120.3 [83.7 ;145.3]	0.61
Foot extensors					
- *Right*	74..3 [72.0 ;88.7]	81.0 [71.7 ;124.7]	126.7 [78.0 ;177.3]	74.3 [65.7 ;109.7]	0.47
- *Left*	65.7 [61.7 ;98.3]	77.0 [60.3 ;135.0]	134.3 [84.0 ;157.3]	80.0 [60.2 ;119.7]	0.20
Foot plantar flexors					
- *Right*	n.a[a]	n.a[a]	n.a[a]	n.a[a]	n.a[a]
- *Left*	123.0 [121.0 ;180.33]	217.7 [151.0 ;280.3]	205.7 [159.3 ;306.0]	235.7 [167.4 ;273.3]	**< 0.01**
PROM (degrees)					
Wrist flexion					
- *Right*	77.5 [70.0 ;90.0]	70.0 [65.0 ;80.0]	72.5 [66.3 ;78.8]	65.0 [60.0 ;83.8]	**0.04**
- *Left*	70.0 [56.3 ;83.8]	65.0 [60.0 ;80.0]	70.0 [61.3 ;83.8]	67.5 [56.3 ;78.8]	0.50
Wrist extension					
- *Right*	90.0 [90.0 ;100.0]	90.0 [90.0 ;100.0]	92.5 [90.0 ;100.0]	92.5 [90.0 ;98.8]	0.68
- *Left*	90.0 [81.25 ;103.8]	90.0 [85.0 ;100.0]	87.5 [81.3 ;100.0]	87.5 [81.3 ;93.8]	0.09
Elbow flexion					
- *Right*	32.5 [21.3 ;38.8]	35.0 [30.0 ;40.0]	35.0 [26.3 ;38.8]	30.0 [30.0 ;33.8]	0.76
- *Left*	32.5 [25.0 ;35.0]	35.0 [30.0 ;35.0]	35.0 [26.3 ;40.0]	30.0 [25.0 ;38.75]	0.54

Elbow extension					
- *Right*	180.0 [176.3 ;180.0]	175.0 [175.0 ;180.0]	177.5 [171.3 ;180.0]	172.5 [170.0 ;178.8]	0.16
- *Left*	180.0 [176.3 ;180.0]	175.0 [170.0 ;180.0]	177.5 [170.0 ;180.0]	175.0 [170.0 ;180.0]	0.22
Hip flexion					
- *Right*	50.0 [42.5 ;58.8]	55.0 [50.0 ;60.0]	57.5 [46.3 ;60.0]	52.5 [45.0 ;67.5]	0.07
- *Left*	50.0 [50.0 ;58.8]	50.0 [50.0 ;60.0]	55.0 [46.3 ;60.0]	55.0 [46.3 ;58.8]	0.35
Hip extension					
- *Right*	10.0 [7.5 ;15.0]	15.0 [10.0 ;15.0]	10.0 [10.0 ;15.0]	10.0 [10.0 ;15.0]	0.75
- *Left*	10.0 [7.5 ;15.0]	15.0 [10.0 ;15.0]	10.0 [10.0 ;15.0]	10.0 [10.0 ;15.0]	0.73
Knee flexion					
- *Right*	37.5 [35.0 ;45.0]	45.0 [40.0 ;50.0]	45.0 [40.0 ;50.0]	47.5 [41.3 ;53.8]	**< 0.01**
- *Left*	42.5 [32.5 ;48.8]	40.0 [40.0 ;50.0]	40.0 [35.0 ;53.8]	47.5 [32.5 ;50.0]	0.15
Knee extension					
- *Right*	180.0 [175.0 ;180.0]	175.0 [175.0 ;180.0]	175.0 [175.0 ;180.0]	175.0 [175.0 ;180.0]	0.19
- *Left*	177.5 [171.3 ;180.0]	180.0 [175.0 ;180.0]	175.0 [175.0 ;180.0]	175.0 [175.0 ;175.0]	0.47
Ankle flexion					
- *Right*	132.5 [98.8 ;162.5]	160.0 [140.0 ;170.0]	160.0 [151.3 ;163.8]	157.5 [142.5 ;163.8]	0.08
- *Left*	125.0 [96.3 ;165.0]	160.0 [155.0 ;160.0]	160.0 [156.3 ;165.0]	160.0 [155.0 ;163.8]	**< 0.01**
Ankle extension					
- *Right*	120.0 [100.0 ;157.5]	95.0 [95.0 ;115.0]	95.0 [91.3 ;98.8]	97.5 [91.3 ;110.0]	0.07
- *Left*	120.0 [95.0 ;153.8]	95.0 [90.0 ;100.0]	95.0 [90.0 ;100.0]	95.0 [90.0 ;102.5]	**0.01**

[a]HHD plantar flexors right: excluded from mixed model since no participants were able to complete this measurement ≥ 2 visits.

SELENON-RM = SELENON-related congenital myopathy; HHD = Hand-held dynamometry. Data is presented as median [p25 ; p75]

Supplementary table 3. Questionnaires LAMA2-MD patients during follow-up.

Questionnaire/domain	Visit 1 (baseline)	Visit 2 (at 6 months)	Visit 3 (at 12 months)	Visit 4 (at 18 months)	P-value
**PedsQL Generic**					
Physical functioning					
Parents proxy	33.7 (±18.3)	30.3 (±14.0)	32.83 (±13.7)	32.8 (±14.0)	0.46
Children's proxy	42.0 (±14.5)	39.9 (±14.9)	41.1 (±20.4)	37.6 (±18.7)	0.14
Emotional functioning					
Parents proxy	76.0 (±17.5)	76.0 (±16.4)	74.3 (±19.4)	79.1 (±14.2)	0.91
Children's proxy	84.1 (±14.1)	81.8 (±11.9)	81.5 (±16.0)	81.4 (±25.9)	0.29
Social functioning					
Parents proxy	67.7 (±14.0)	66.7 (±13.5)	64.6 (±14.1)	67.3 (±13.3)	0.52
Children's proxy	63.6 (±19.5)	72.3 (±11.3)	74.5 (±14.4)	74.1 (±15.6)	**0.03**
School functioning					
Parents proxy	74.6 (±14.7)	72.5 (±13.3)	66.8 (±17.9)	78.4 (±11.1)	0.85
Children's proxy	71.4 (±12.3)	75.9 (±14.6)	77.0 (±10.1)	74.6 (±16.7)	0.12
**PedsQL NMM**					
About neuromuscular disease					
Parents proxy	56.7 (±16.7)	59.63 (±12.9)	57.7 (±15.0)	60.1 (±12.4)	0.75
Children's proxy	73.0 (±10.5)	68.3 (±13.0)	72.2 (±11.9)	67.5 (±17.3)	0.87
Communication					
Parents proxy	76.4 (±21.4)	76.4 (±20.4)	80.7 (±16.6)	79.9 (±14.1)	0.63
Children's proxy	90.6 (±11.3)	87.0 (±14.5)	86.5 (±18.3)	86.5 (±25.6)	0.71
Family resources					
Parents proxy	70.6 (±12.8)	76.7 (±13.5)	76.1 (±15.1)	76.4 (±10.9)	0.21
Children's proxy	86.3 (±13.0)	86.1 (±12.7)	86.9 (±10.7)	88.1 (±10.3)	0.64
**PedsQL MFS**					
General Fatigue					
Parents proxy	65.7 (±14.2)	70.3 (±14.0)	68.5 (±12.3)	71.2 (±10.8)	0.52
Children's proxy	77.4 (±13.6)	77.8 (±12.1)	81.1 (±21.28)	86.4 (±16.3)	0.82
Sleep/rest Fatigue					
Parents proxy	83.9 (±12.3)	83.7 (±11.8)	86.3 (±9.2)	80.3 (±9.9)	0.17
Children's proxy	75.7 (±12.9)	82.3 (±12.8)	82.6 (±10.3)	83.7 (±8.2)	**0.03**
Cognitive Fatigue					
Parents proxy	79.9 (±19.2)	75.5 (±13.3)	79.8 (±13.6)	82.1 (±11.7)	0.92
Children's proxy	73.3 (±20.8)	75.4 (±13.6)	81.4 (±12.4)	81.8 (±16.0)	0.06
**CIS**					
Activity	13.4 (±3.3)	12.9 (±2.6)	12.9 (±2.4)	12.6 (±2.3)	0.54
Concentration	20.6 (±3.3)	21.0 (±2.3)	20.6 (±2.9)	19.8 (±3.1)	0.27
Motivation	13.1 (±2.8)	15.0 (±3.1)	13.2 (±3.4)	13.6 (±2.2)	0.84
Fatigue	32.6 (±4.6)	34.0 (±5.8)	32.9 (±4.0)	33.5 (±4.9)	0.70
**EK2**	20.7 (15.6)	22.1 (14.7)	19.5 (14.0)	17.1 (14.6)	0.06
**McGill questionnaire**	6.6 (2.7)	5.3 (2.7)	5.8 (2.2)	6.3 (3.1)	0.997
**Wong-Baker faces pain rating scale**					
Currently	0.3 (0.5)	0.4 (0.9)	0.2 (0.4)	0.3 (0.6)	0.52
Best of the week	0.1 (0.3)	0.2 (0.6)	0.2 (0.5)	0.2 (0.4)	0.19
Worst of the week	0.6 (1.0)	0.6 (0.9)	0.4 (0.7)	0.6 (1.1)	0.96

LAMA2-MD = LAMA2-related muscular dystrophy; PedsQL = Pediatric quality of life inventory; NMM = Neuromuscular Module; MFS = Multidimensional Fatigue Scale; CIS = Checklist individual strength; EK2 = Egen Klassification version 2. Data is presented as mean ± SD.

Supplementary table 4. Questionnaires SELENON-RM patients during follow-up.

Questionnaire/domain	Visit 1 (baseline)	Visit 2 (at 6 months)	Visit 3 (at 12 months)	Visit 4 (at 18 months)	P-value
**PedsQL Generic**					
Physical functioning					
Parents proxy	36.1 (±21.0)	35.4 (±17.8)	36.8 (±17.4)	30.6 (±16.2)	0.73
Children's proxy	41.9 (±26.8)	48.1 (±31.8)	42.5 (±29.0)	34.4 (±20.9)	0.89
Emotional functioning					
Parents proxy	60.8 (±13.7)	63.8 (±9.2)	67.5 (±11.5)	59.0 (±7.6)	0.82
Children's proxy	67.0 (±12.6)	74.0 (±16.7)	62.0 (±2.7)	62.5 (±23.3)	0.45
Social functioning					
Parents proxy	62.1 (±10.3)	65.8 (±11.5)	68.8 (±11.3)	59.0 (±13.3)	0.82
Children's proxy	57.0 (±12.0)	80.0 (±12.8)	72.0 (±13.5)	65.0 (±16.8)	0.40
School functioning					
Parents proxy	59.7 (±11.8)	65.0 (±16.0)	69.3 (±14.9)	63.5 (±15.9)	0.18
Children's proxy	65.0 (±14.1)	62.0 (±25.9)	57.0 (±11.5)	52.5 (±13.2)	0.15
**PedsQL NMM**					
About neuromuscular disease					
Parents proxy	52.8 (±9.6)	54.7 (±11.9)	57.5 (±14.4)	51.2 (±6.6)	0.34
Children's proxy	56.8 (±17.4)	63.5 (±24.9)	60.0 (±20.1)	56.6 (±18.5)	0.56
Communication					
Parents proxy	59.0 (±34.1)	68.1 (±26.3)	74.3 (±12.2)	73.3 (±17.3)	0.07
Children's proxy	60.0 (±19.0)	76.7 (±25.3)	61.7 (±17.3)	60.4 (±33.6)	0.75
Family resources					
Parents proxy	63.3 (±6.6)	67.1 (±10.5)	71.3 (±3.1)	68.5 (±7.0)	0.06
Children's proxy	80.0 (±12.7)	83.0 (±20.5)	76.0 (±19.2)	80.0 (±21.6)	0.94
**PedsQL MFS**					
General Fatigue					
Parents proxy	47.1 (±19.6)	46.7 (±22.4)	49.6 (±20.0)	45.3 (±8.6)	0.53
Children's proxy	50.0 (±30.6)	57.6 (±27.1)	54.2 (±24.0)	51.7 (±17.8)	0.69
Sleep/rest Fatigue					
Parents proxy	67.4 (±10.8)	79.8 (±13.5)	79.9 (±14.9)	76.5 (±10.8)	0.44
Children's proxy	64.6 (±30.9)	72.2 (±27.1)	68.1 (±25.0)	63.3 (±24.5)	0.74
Cognitive Fatigue					
Parents proxy	60.8 (±20.3)	66.7 (±25.4)	68.3 (±23.7)	60.9 (±10.5)	0.08
Children's proxy	59.0 (±17.4)	68.8 (±25.9)	66.0 (±20.8)	73.3 (±14.9)	0.95
**CIS**					
Activity	12.8 (±2.3)	11.6 (±1.8)	12.0 (±2.1)	11.6 (±1.8)	0.07
Concentration	21.2 (±2.4)	22.8 (±1.3)	23.8 (±0.8)	21.6 (±0.9)	0.54
Motivation	17.0 (±2.9)	17.2 (±3.3)	17.4 (±4.0)	17.0 (±3.1)	0.95
Fatigue	34.6 (±3.0)	30.6 (±3.4)	29.6 (±4.0)	30.6 (±2.1)	**0.01**
**EK2**	7.8 (8.4)	6.8 (7.3)	6.0 (5.4)	8.1 (8.0)	0.83
**McGill**	7.3 (2.3)	8.0 (1.5)	7.3 (3.6)	6.8 (3.3)	0.90
**Wong-Baker faces pain rating scale**					
Currently	0.9 (1.1)	0.6 (1.1)	1.1 (1.1)	0.6 (0.8)	0.69
Best of the week	0.5 (0.7)	0.2 (0.4)	0.2 (0.4)	0.3 (0.7)	0.29
Worst of the week	2.1 (1.7)	1.2 (1.1)	1.6 (0.9)	1.3 (1.2)	0.16

SELENON-RM = SELENON-related congenital myopathy; PedsQL = Pediatric quality of life inventory; NMM = Neuromuscular Module; MFS = Multidimensional Fatigue Scale; CIS = Checklist individual strength; EK2 = Egen Klassification version 2. Data is presented as mean ± SD.

Supplementary figures

Figure 1. Hand-held dynamometry per muscle in LAMA2-MD patients during follow-up

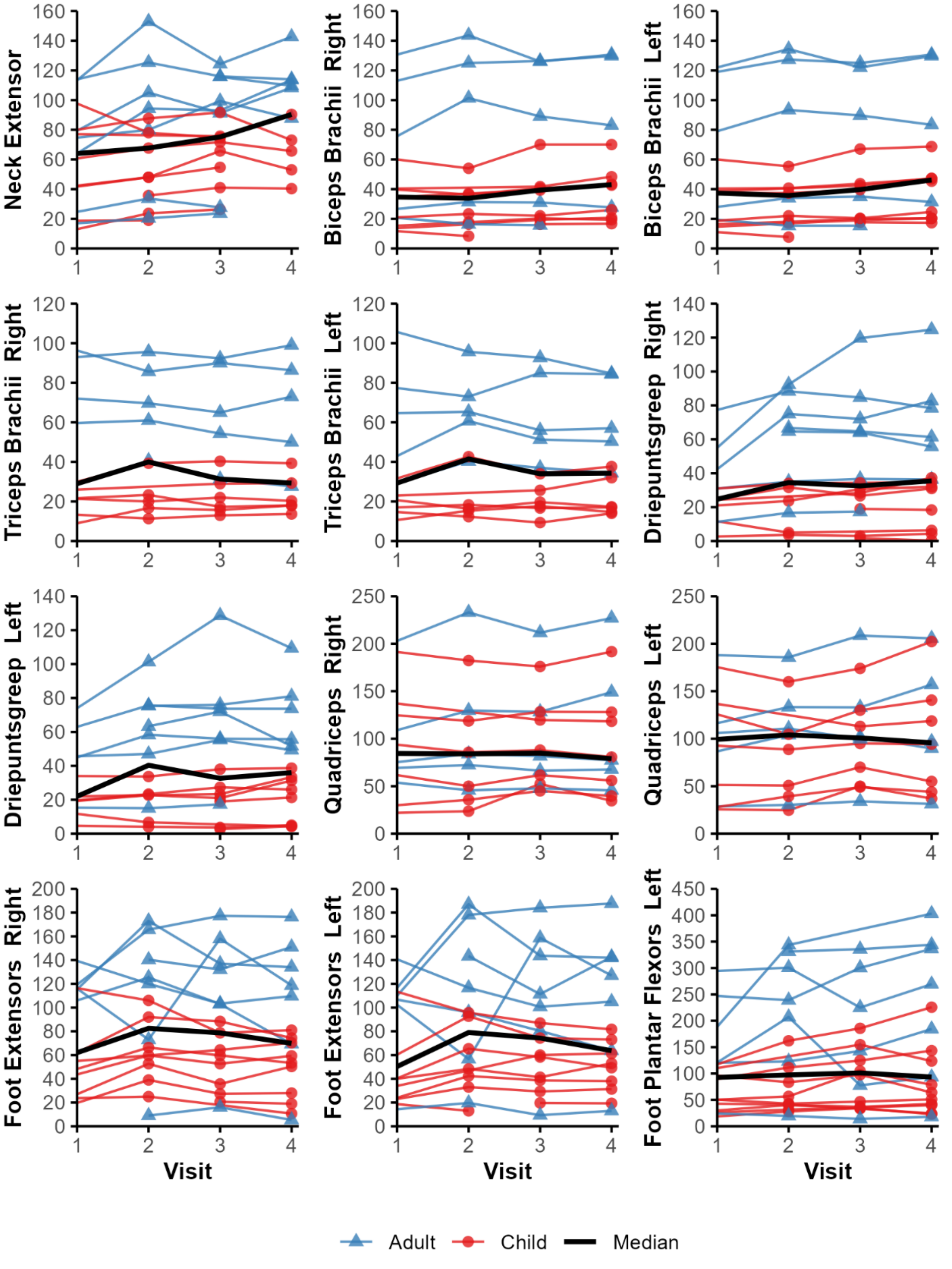

LAMA2-MD = LAMA2-related muscular dystrophy. Y-axis represents muscle strength, expressed in Newtons (N).

Figure 2. Hand-held dynamometry per muscle in SELENON-RM patients during follow-up

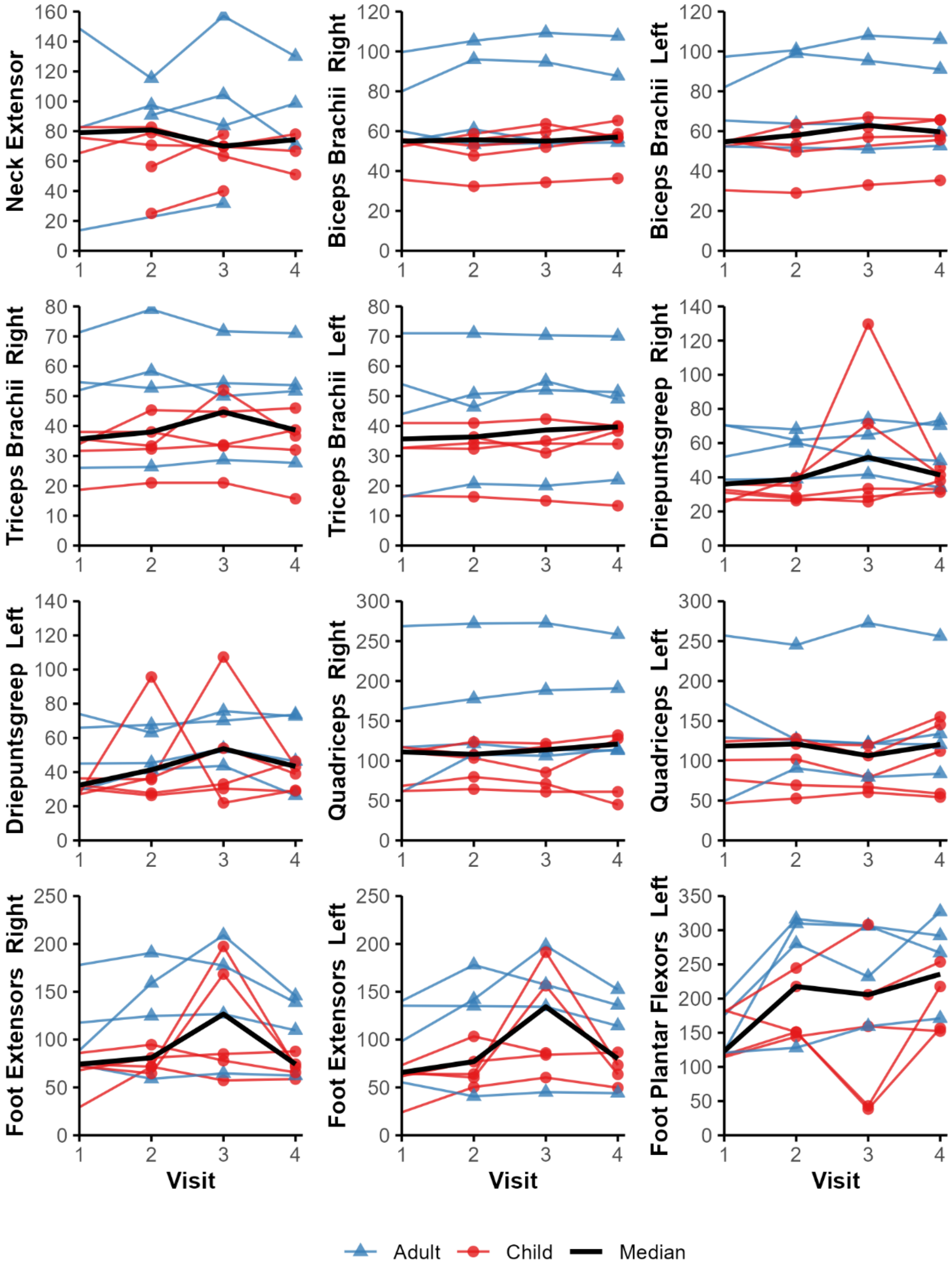

SELENON-RM = SELENON-related congenital myopathy. Y-axis represents muscle strength, expressed in Newtons (N).
